# Efect of periodontal disease and non surgical periodontal treatment on C-reactive protein. Evaluation of type 1 diabetic patients

**DOI:** 10.4317/medoral.17793

**Published:** 2012-02-09

**Authors:** Fernando Llambés, Francisco J. Silvestre, Antonio Hernández-Mijares, Rami Guiha, Daniel Bautista, Raúl Caffesse

**Affiliations:** 1 DDS, PhD. University of Valencia (Spain); 2 DDS, MD, PhD. Department of Stomatology. Dr. Peset Hospital Valencia (Spain); 3 MD, PhD. Department of Endocrinology Dr. Peset Hospital, Valencia (Spain); 4 DDS, MS, PhD. Misr International University, Cairo, (Egypt); 5 MD, PhD. Department of Epidemiology. Dr. Peset Hospital, Valencia (Spain); 6 DDS, MS, PhD. Department of Periodontics. Complutense University, Madrid (Spain)

## Abstract

Objectives: The purpose of this study was to analyze how anti-infectious periodontal treatment affects C reactive protein (CRP) values in patients with type 1 diabetes, and correlate baseline CRP levels with periodontal disease severity. Study Design: A cohort of fifty three subjects with type 1 diabetes and moderate to severe periodontitis were recruited. Periodontal parameters were measured, and blood samples were obtained to evaluate high-sensitivity C-reactive protein (hs-CRP). Group 1 was treated with scaling, root planning, and systemic administration of doxycycline. Group 2 received only scaling and root planning. Results: Hs-CRP was reduced after periodontal treatment in group 1 (-0.22 mg/l) and 2 (-0.21 mg/l ) but this reduction was not statistically significant, even in the patients with the best response to periodontal treatment. However, significant correlation appeared between hs-CRP and mean probing pocket depth (PPD) (p=0, 01) and mean clinical attachment level (CAL) (p=0,03). Conclusions: Non-surgical periodontal treatment couldn’t reduce hs-CRP values, however, it was found an association between advanced periodontitis and elevated blood hs-CRP levels in patients with type 1 diabetes. It can be speculated that periodontal disease increases production of pro-inflammatory mediators in patients with type 1 diabetes, but other producing sources of these pro-inflammatory substances may exist.

** Key words:**Periodontal disease, periodontitis, diabetes mellitus type 1, periodontal therapy, C reactive protein.

## Introduction

Periodontal disease is a chronic infectious disease, triggered by the bacterial biofilm of dental plaque, which produced an inflammatory destruction of soft and hard tooth supporting structures (1). It is a high prevalence disease and studies have shown that about 10% of the adult population and about 30% of individuals over the age of 50 years suffer from severe periodontitis. This prevalence should be even higher in patients with diabetes because clinical and epidemiological studies have reported that patients with a long history of diabetes seem to have more periodontal tissue breakdown than age matched, non-diabetic controls (2,3). Severity and progression of the periodontitis is also higher in individuals with diabetes (4).

The host responds to the periodontal infections through the immune system which produce inflammatory mediators after the interaction with the bacteria plaque. Although periodontitis is a chronic infection, acute-phase elements are also involved in this immunological response and confirm that in periodontitis a systemic inflammation is present (5). This acute-phase reactants have pro-inflammatory properties; they help to neutralize invasive pathogens and stimulate repair and regeneration of tissues. C-reactive protein (CRP), plasminogen-activator 1(PAI-1), and fibrinogen are acute-phase reactants, but among all of them, CRP in particular has been focus of attention because elevated levels constitute a risk factor for cardiovascular disease (CVD) (6,7). Nowadays, CRP is considered as a biomarker of systemic inflammation.

Diabetes Mellitus is a multi factorial metabolic disease. Recent evidence suggests that chronic sub clinical inflammation plays an important role in the development of the disease (8). On the other hand, elevated circulating levels of sub clinical inflammatory markers, such as C-reactive protein (CRP) and interleukin-6 (IL-6) are reported to be significant risk indicators not only for the development of cardiovascular disease, but also for the development or progression of diabetes (9). Several reports have demonstrated elevated serum CRP levels in patients with periodontitis compared with healthy controls (10,11) and some clinical studies have suggested that successful non-surgical periodontal treatment can reduce CRP levels in systemically healthy subjects (12). However these results are inconclusive (13,14) and only a few articles in the literature analyze the relationship between CRP and periodontal disease in patients with diabetes and none of them are done with type 1 diabetic individuals (15-17).

The purpose of this study was to analyze how anti-infectious periodontal treatment affects CRP values in patients with type 1 diabetes and if baseline CRP levels are related to periodontal disease severity. Our results will help to understand the role of plasma inflammatory mediators in subjects with diabetes as well as their relationship with the periodontal disease and its treatment. We have to keep in mind that high CRP values could increase the risk for the development and progression of diabetes, and if they would be reduced, a better control of diabetes and periodontal disease could be achieved.

## Material and Methods

Study population

This randomized controlled clinical pilot study was conducted at Dr. Peset University Hospital in Valencia (Spain). Study protocol was approved by Dr. Peset Hospital Clinic Research Committee, and participants signed an approved consent form to participate in the study. Fifty three subjects with type 1 diabetes and moderate to severe periodontitis (25 females and 28 males) ranging in age from 19 to 60 (mean 35 ± 9 years) were recruited from the endocrinology division for this single-blind study. Patients with type 1 diabetes were diagnosed according to the criteria published by the American Diabetes Association in 1997 (18), and they were treated by insulin, diet, and physical exercise recommendations.

Participants had diabetes for more than 1 year, and none of them had other major illnesses or severe diabetic complications. Patients had not taken antibiotics for at least 3 months prior to baseline and did not have any active infection. A panoramic radiograph was taken to assure that neither extensive caries nor periapical lesions were present. Eligible subjects had 14 or more natural teeth, of which at least five had a site with probing pocket depth (PPD) ≥ 5 mm and clinical attachment level (CAL) ≥ 3 mm. From this point, subjects with moderate to severe periodontal disease were included. They had not had periodontal treatment or professional cleaning of the teeth for at least 1 year prior to the study. Pregnant and breast feeding women were excluded. Twenty patients (38%) had good diabetic control (HbA1c < 7%), 12 individuals (22%) had moderate control (HbA1c between 7% and 8%), and 40% of the sample (21 diabetic individuals) had poor metabolic control (HbA1c > 8%) according to the American Diabetes Association criteria (19). Most of the patients selected were nonsmokers (32 patients), some smoked less than 15 cigarettes per day (11 patients), and the rest were heavy smokers consuming 15 or more cigarettes per day (10 patients) ( Table 1).

Laboratory and periodontal examination

**Table 1:**
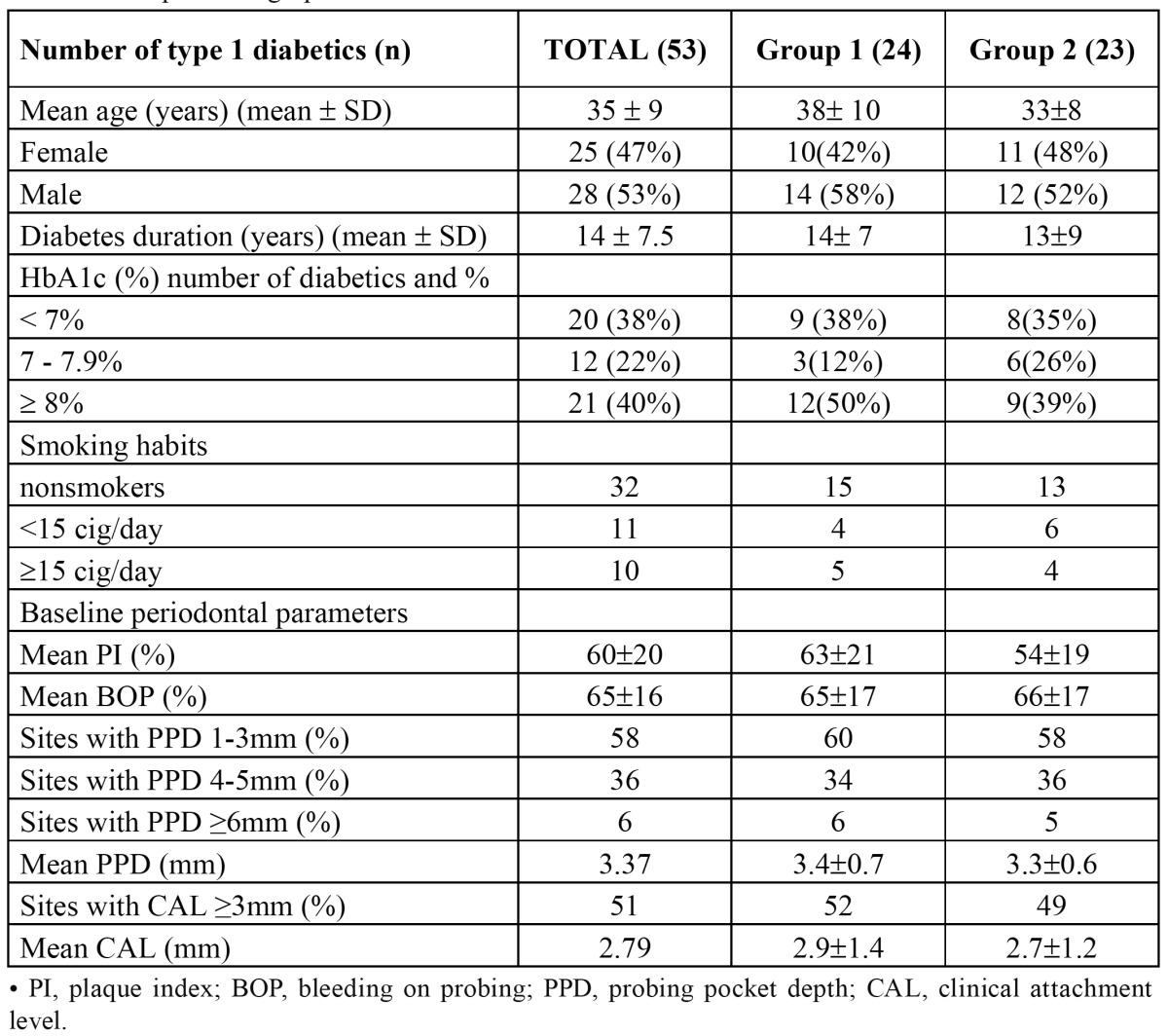
Sample Demographics.

Fasting venous blood was collected in vacuum tubes early in the morning, and high-sensitivity C-reactive protein (hs-CRP) was measured. Plasma hs-CRP levels were assessed using a kit with specific high-sensitivity methodology in a spectrophotometer according to the manufacturer’s instructions. The test samples were treated with a specific antibody to human CRP in a suitable buffer. The turbidity induced by the formation of immune complexes was measured at 546 nm, and the values were automatically calculated from a known standard. The lower detection limit for the assay was 0.1 mg/l. A commercial control serum was used to verify the assay performance.

Patients also received an oral soft tissue examination including periodontal measurements of plaque index (PI), bleeding on probing (BOP), PPD, and CAL for all teeth present. O’Leary PI was measured in four areas per tooth (mesiobuccal, midbuccal, distobuccal, and midlingual) (20), and the other periodontal parameters were registered on six sites by tooth (mesiobuccal, midbuccal, distobuccal, mesiolingual, midlingual, and distolingual).

Study design

All patients with type 1 diabetes from Dr. Peset Hospital were screened (One hundred thirty-six), and 72 were found to match the selection criteria for this pilot study. They were told not to change their diet, exercise, or insulin dose unless absolutely necessary and to inform investigators if any change occurred. Hs-CRP values at the screening time, 3 month previous to the beginning of the study, were obtained from the medical records of the patients to evaluate changes in this parameter before the initiation of the investigation. These hs-CRP values were obtained in the same center and were made by the same laboratory that was used for this clinical protocol. The sample was randomized, allowing the subjects to self-select a coded number contained in an envelope; this number identified the group to which the patient was assigned (group 1 or 2). Baseline examination was performed 3 months after screening and within the 30 days prior to the beginning of the periodontal treatment. All periodontal measurements were taken by only one trained periodontist. Intra-examiner reproducibility was calculated and showed that periodontal clinical attachment measurements were in agreement within 2 mm more than 90% of the time. This clinician was blinded to the treatment applied in each patient and care was taken that subjects did not disclose their group category (21).

Group 1 had baseline Hs-CRP measured just before the beginning of the periodontal treatment. Subjects were instructed on the modified Bass brushing technique and inter proximal cleaning. After that, scaling and root planning (SRP) under local anesthesia was performed by two trained dental hygienists using ultrasonic devices and manual Gracey curets (Hu-Friedy®, Chicago USA). SRP was scheduled in one or two sessions 1 week apart according to the periodontal disease severity and the number of teeth present. No less than 30 min were assigned to each quadrant. Chlorhexidine rinses 0,2% were prescribed after SRP (20 ml during 30 s, twice daily) and maintained for 12 weeks to the end of the clinical protocol. No other rinses or toothpaste was used during the study. Individuals were placed on doxycycline 100 mg (b.i.d. for the first day and then one capsule per day thereafter) for 15 days.

Group 2 had the same treatment as group 1 with the exception of the doxycycline which was not used in this group.

Twelve weeks after treatment, blood samples were taken again, and hs-CRP was analyzed. At the same time, periodontal parameters were measured again. Periodontal surgical treatment was recommended to patients with probing pocket depths ≥6mm. Compliance with use of oral hygiene devices, chlorhexidine, and doxycycline was assessed with a personal oral interview with the participants. It was classified as good (instructions were followed), fair or poor (prescriptions were not followed).

At the end of the study, 19 subjects were dropped out. Nine patients did not follow postoperative anti-infectious treatment (clorhexidine rinses and doxycycline if prescribed), 3 subjects had active acute infections during post-treatment period and 7 patients had an inadequate baseline laboratory exam (high-sensitivity C-reactive protein test could not be performed). Finally, baseline hs-CRP could be obtained from 53 patients and post-treatment hs-CRP was measured in 47 individuals (24 patient from group 1 and 23 patients from group 2).

Statistical analysis

First, laboratory and periodontal data were analyzed for distribution. Kolmogorov-Smirnov test showed that hs-CRP values before and after periodontal treatment (primary variables) did not follow a normal distribution. Consequently, hs-CRP data was expressed as median with minimum and maximum and non-parametric statistics were used to analyze the results. Periodontal parameters (secondary variables) followed a normal distribution and mean with standard deviation values were given.

Hs-CRP changes after non surgical periodontal treatment were studied by using the Wilcoxon test, and differences were considered significant when p<0.05. Power analysis was performed.

Baseline correlations between non-parametric hs-CRP values and periodontal measurements were analyzed with the Spearman test. Correlations were considered statistically significant when p<0.05. Correlation between hs-CRP and independent variables such as age, sex, diabetes duration, diabetes control, diabetes complications and smoking habits was performed with the same test.

## Results

Baseline Hs-CRP was obtained from 53 type 1 diabetic subjects who participated in the study and was expressed in mg/l. Sample description has been reported previously. This data was correlated with periodontal clinical parameters on each patient such as percentages of BOP sites, mean PPD and mean CAL. No correlation was found between hs-CRP and BOP (r-squared=0.12 and p=0.39), however, significant correlation appeared between hs-CRP and mean PPD (r-squared= 0.33 and p=0, 01) and between hs-CRP and mean CAL (r=0.29 and p=0,03). Higher values of hs-CRP were associated with higher PPD and more CAL. These results show that higher hs-CRP is found as periodontal disease severity increase. Statistical analysis did not show any correlation between hs-CRP and other variables such as age, sex, diabetes duration, diabetes control, diabetes complications and smoking habits which exclude these factors as confounders.

Hs-CRP value at the screening time was compared with hs-CRP at baseline, just before periodontal treatment was performed 3 months after screening. The goal of this analysis was to observe how hs-CRP changed in 3 months in the absence of dental treatment. Hs-CRP could be obtained from the medical records of 17 patients and it was observed a mean absolute oscillation of 0.5 mg/l during this time frame. The minimum change experienced was 0%, and the maximum change was 3.8%. Mean hs-CRP and standard deviation could not be calculated because sample had not a parametric distribution.

Baseline periodontal characteristics of the sample can be observed in ( Table 1). Patients have a moderate to severe periodontal disease (mean PPD=3.37mm and mean CAL=2.79mm) with significant BOP (mean BOP=60%) and no good plaque control (mean PI=60%). These patients were divided in two groups. Group 1 (24 subjects) were treated with non surgical periodontal treatment associated to doxycycline for 15 days. Group 2 (23 individuals) were treated as group 1 with the exception of doxycycline. Baseline data across all clinical periodontal parameters was almost within the same range and statistical analysis did not show any significant difference between the two groups at baseline visit (p > 0.05). Both groups showed very good periodontal response to treatment; PI, BOP, PPD, and CAL showed a very significant improvement. However, after this successful non surgical periodontal treatment, hs-CRP levels did not change significantly.

Hs-CRP changes after periodontal treatment were analyzed in group 1 and in group 2. Median hs-CRP at baseline was 1.27mg/l (min0.16-max7.6) in group 1and 1.19 (min0.16-max5.4) in group 2. Three months after treatment, median hs-CRP was 1.05mg/l (min0.16-max7.8) and 0.98 (min0.16-max5.4) respectively. These differences were not statistically significant according to Wilcoxon test ( Table 2). This sample had a statistical power of 86% to detect hs-CRP changes after periodontal treatment ≥0.5mg/l (p<0.05). No difference between groups was observed comparing hs-CRP reduction after periodontal treatment.

**Table 2:**
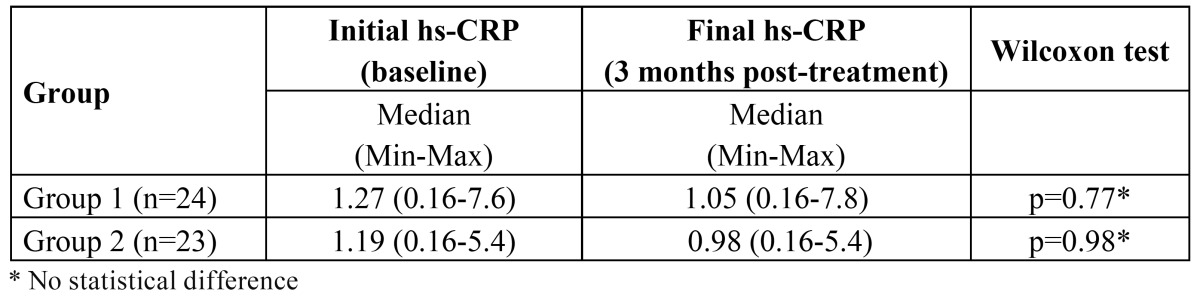
hs-CRP values (mg/l) at baseline and 3 months after treatment. Median (Minimum-Maximum) are presented.

Hs-CRP variations were also studied in the patients with the best periodontal response to treatment ( Table 3). To achieve this goal, patients with the highest BOP and PPD reduction after periodontal treatment were selected from group 1 and 2. A total of 17 patients with BOP reduction above 60% was obtained; nine of these patients came from group 1, and 8 patients came from group 2. In this group of patients with the best BOP reduction, median hs-CRP was 1.3 mg/l (min0.2-max5.5) initially and 0.92 mg/l (0.2min-max7.8) at the re-evaluation. Reduction of hs-CRP after periodontal treatment was not statistically significant (p=0.09). Another group was made with patients with the most significant PPD reduction after therapy. For this purpose, 24 patients were selected, and these patients had reduced the mean baseline PPD to 18% or more. Thirteen of these patients were from group 1 and eleven were from group 2. Median hs-CRP was 1.43 mg/l (min0.2-max7.6) at baseline and 1.38 mg/l (min0.2-max7.8) after treatment, which showed a post-treatment minimal reduction on hs-CRP value that was not statistically significant (p=0.75).

**Table 3:**
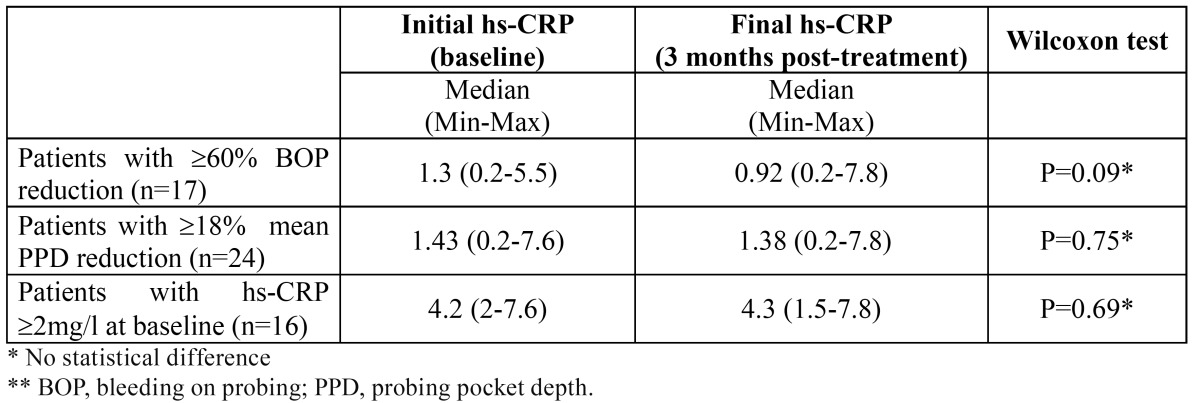
hs-CRP values (mg/l) at baseline and 3 months after treatment in the subgroups with the best periodontal response to treatment and with the higher hs-CRP at baseline. Median (Minimum-Maximum) are presented.

Patients with diabetes and the higher hs-PRC at baseline were selected from groups 1 and 2, and hs-CRP oscillations were analyzed. Sixteen patients with initial hs-CRP ≥ 2 mg/l were included, 7 from group 1 and 9 from group 2. Median hs-CRP was 4.2mg/l (min2-max7.6) initially and 4.3 mg/l (min 1.5-max7.8) 3 months after periodontal treatment, and these minimal changes were not significant (p=0.69). These results suggest that hs-CRP might not change after periodontal treatment in patients with type 1 diabetes, even in the cases with the highest initial hs-CRP ( Table 3).

Differences between smokers and nonsmokers regarding hs-CRP at baseline and after periodontal treatment were not analyzed in this study because the smokers group was very small. Only 10 patients from the total sample smoked ≥15 cigarettes a day. Thus, statistical analysis of this comparison was not possible.

## Discussion

Eligible subjects that participate in this study, had 14 or more natural teeth, of which at least five had a site with probing pocket depth (PPD) ≥ 5 mm and clinical attachment level (CAL) ≥ 3 mm. We have found in the literatures different criteria to evaluate periodontal disease severity and a consensus cannot be established. Therefore, we studied patients with at least 5 teeth with one PPD ≥ 5mm and CAL ≥ 3 mm, to assure that some degree of periodontal destruction and periodontal inflammation was present in the sample.

This clinical study has found a correlation between advanced periodontitis and elevated blood hs-CRP levels in patients with type 1 diabetes, however, periodontal treatment could not reduce significantly hs-CRP values, even in the patients with the best response to this treatment.

Mean PPD and mean CAL is a continuous variable to evaluate periodontal disease severity (22), other authors have used discontinuous variables classifying this disease in low threshold periodontitis (mild periodontal disease) and high threshold periodontitis (advanced disease) (23). Mean PPD and mean CAL are a pretty accurate ways of evaluating periodontal disease severity and these values were correlated with hs-CRP levels to find any relationship. After analyzing correlations, results have shown that higher values of hs-CRP are found as periodontal disease severity increase. This finding could be expected because several reports have indicated how periodontal disease acts as any other chronic infection or inflammatory process in the body. Periodontitis produces bacteraemias (24,25) and the host responds with elevated levels of interleukin-6 (IL-6), known to induce hepatocytes to produce CRP and other acute-phase proteins and pro-coagulant mediators (5). Thus, it is not surprising that high levels of CRP have been observed in peripheral blood samples in patients with type 1 diabetes and severe periodontitis. Other authors have found the same association in healthy individuals (10,11,26,27).

A correlation between mean BOP and hs-CRP was not found and this could be explained considering that BOP can be produced by periodontitis but also by plaque induced gingivitis and consequently, this is not a good parameter to evaluate periodontal disease severity.

High levels of CRP are not recommended in subjects with type 1 diabetes because they can increase insulin resistance and they can also exacerbate ongoing inflammatory processes in the blood vessels accelerating the progression of diabetic complications. It has been shown that elevated CRP levels in patients with periodontitis affects inflammation in atherosclerotic lesions, increasing the risk for cardiovascular and cerebrovascular events (28,29). High CRP levels (42.1 mg/l) are associated with a higher incidence of acute thrombotic events including stroke and myocardial infarction (29).

Non surgical periodontal treatment performed in this clinical study was very effective in reducing periodontal inflammation and these results has been report previously (22). This periodontal improvement was followed by a reduction on hs-CRP values but not to a significant level.

The sample studied had a statistical power of 86% to detect hs-CRP changes after periodontal treatment ≥0.5mg/l (p<0.05) which could be considered clinically significant. The hs-CRP reduction after periodontal treatment detected in this study could be statistically significant increasing the sample size, but this 0.2mg/l reduction may not be clinically significant.

Even in the patients with the best response to periodontal treatment reduction was not statistically significant. Neither diabetic patient with the higher initial hs-CRP show any change in this parameter after periodontal treatment. Long-term clinical studies evaluating the effect of periodontal treatment on CRP values are scarce, especially when patients with diabetes are considered. Some treatment studies on healthy individuals have shown a reduction on CRP levels after periodontal treatment (12). In two studies from D’Aiuto et al. (30) a standard and an intense periodontal treatment was given to different treatment groups. Both treatment modalities reduced blood CRP levels but the meta-analysis comparing the two treatment protocols (standard versus intensive treatment) failed to show a significant benefit of the intensive treatment, however a trend towards intensive treatment was noted. There are also reports in the literature on patients with type 2 diabetes that found no clear effect of periodontal treatment on CRP (16,17) and same results can be found in healthy patients with periodontal disease (13,14).

We have to consider that CRP is a nonspecific marker of the acute-phase response, and according to that, periodontal disease but also other potential stimuli such as unknown chronic infections or inflammatory conditions may produce mild CRP increases. Some authors have shown that smoking, obesity or trauma may affect CRP values (31) and we also have to keep in mind that vascular alteration in diabetic patients are inflammatory processes that could increase blood inflammatory mediators. On the other hand, it has been reported that a hyper-reactive immune system is associated with periodontal disease. According to this, it could be speculated that certain diabetic individuals would have a more hyper-reactive immune system, which would produce more periodontal destruction, but also a higher blood concentration of inflammatory mediators as a response to the damage of the circulatory system that diabetes mellitus produces. Thus, periodontal treatment would reduce periodontal inflammation significantly but not blood inflammatory substances such as hs-CRP.

It is concluded that there was an association between advanced periodontitis and elevated blood hs-CRP levels in patients with type 1 diabetes. Non-surgical periodontal treatment could not reduce hs-CRP values to a significant level. It can be speculated that periodontal disease increases production of pro-inflammatory mediators such as CRP in subjects with type 1 diabetes but other producing sources of these pro-inflammatory substances should be identified in these patients. These high CRP levels could be involved in the development of diabetic complications and periodontal disease progression and reduction of these values should be a medical goal in these patients. More studies increasing sample size are needed to clarify the benefits of periodontal treatment in patients with type 1 diabetes and to relate inflammatory mediators with periodontal progression and diabetic complications.
